# Prevalence, serotypes, and antimicrobial resistance of *Salmonella* isolates from patients with diarrhea in Shenzhen, China

**DOI:** 10.1186/s12866-020-01886-5

**Published:** 2020-07-06

**Authors:** Hongwei Shen, Haochuan Chen, Yongxuan Ou, Tingting Huang, Siping Chen, Lintao Zhou, Jinjin Zhang, Qinghua Hu, Yiwen Zhou, Wen Ma

**Affiliations:** 1 Shenzhen Hospital, Southern Medical University, Xinhu Road 1333, Baoan District, Shenzhen, 518110 Guangdong China; 2 Futian District Center for Disease Control and Prevention, Hongli Xilu 8043, Futian District, Shenzhen, 518040 China; 3 Shenzhen Children’s Hospital, Yitian Road 7019, Shenzhen, 518038 China; 4 Shenzhen Center for Disease Control and Prevention, Longyuan Road 8, Nanshan District, Shenzhen, 518000 China

**Keywords:** *Salmonella*, Prevalence, Serotype, Antimicrobial resistance, Diarrhea

## Abstract

**Background:**

*Salmonella* is one of the main causative agents of diarrhea which results in substantial disease burden. To determine the prevalence, serotype distribution, and antimicrobial resistance profiles of clinical *Salmonella* isolates in Shenzhen, a 6-year surveillance study was conducted.

**Results:**

A total of 297 (5.7%) *Salmonella* strains were isolated from stool samples from 5239 patients. Among the 42 serotypes identified, serotype Typhimurium was the most common one which represented 39.7% of the isolates (118), followed by serotype Enteritidis (71, 23.9%), London (12, 4.0%), 4, 5, 12: i: - (11, 3.7%), and Senftenberg (8, 2.7%). A high frequency of resistance was found in ampicillin (70.6%), piperacillin (64.5%), tetracycline (63.5%), and streptomycin (54.3%). Resistance to ampicillin and tetracycline was observed in 95.3% of *S.* Typhimurium isolates; and nalidixic acid in 93.1% of *S.* Enteritidis isolates. Resistance to 5 or more antimicrobial agents was found in 78.8% of *S.* Typhimurium and 69.0% of *S.* Enteritidis isolates. A decreased susceptibility to ciprofloxacin and levofloxacin was associated with amino acid alteration in *gyrA* gene. Point mutations without amino acid changes were seen in *gyrB, parC,* and *parE* genes.

**Conclusions:**

A broad range of serotypes are responsible for Salmonellosis in Shenzhen, with Enteritidis and Typhimurium being the most common serotypes. The high level of antibiotic resistance is of public health significance and ongoing monitoring combined with rational use of antibiotics are recommended. Point mutations in *gyrA* gene might play an important role in the resistance to fluoroquinolones.

## Background

*Salmonella* is a main foodborne and waterborne pathogen worldwide which causes an annual death of 230,000 [1]. *Salmonella-*associated foodborne outbreaks were transmitted by contaminated food such as beef, pork, tomato, and cucumbers [2–5]. It was prevalent throughout the year, but was the most commonly detected between April and October in China [6]. Children less than 5 years old accounted for the largest proportion of infections [7, 8].

Over 2500 serotypes have been reported [9], with Enteritidis and Typhimurium being the most common serotypes causing gastroenteritis [10]. *Salmonella* isolates with different serotypes vary in the pathogenicity, prevalence, and sensitivity to antibiotics. *Salmonella* Typhi and *Salmonella* Paratyphi are usually associated with higher mortality [11]. Some serotypes were reported in only single region of the world, such as *Salmonella* Rissen and *Salmonella* Weltevreden were only identified in Asia [12, 13]. Multi-drug resistance was the most common in *S*. Typhimurium [14].

Salmonellosis is not a severe infection, but the emergence of third-generation cephalosporins and multi-drug resistant isolates has raised concerns [15, 16]. The wide use of antibiotics in poultry and for empirical treatment of salmonellosis has led to the rising of drug resistance rate of *Salmonella*. A high resistance rate to at least one class of clinically important antimicrobials including quinolones was found in the clinical and animal-derived (chicken and pork) isolates [16, 17]. A wide range of mechanisms were associated with cephalosporins and quinolone resistance, including mutation in the quinolone-resistance determining regions (QRDRs), over-expression of an efflux pump and acquisition of drug resistance plasmids [18].

A one-year surveillance was conducted in our previous research and *Salmonella* was found one of the main causes (12.1%) of acute infectious diarrhea in Shenzhen [19]. Nevertheless, the long-term trend of serotype distribution and antimicrobial resistance pattern of the *Salmonella* isolates were not defined. To improve our understanding of the prevalence of *Salmonella* in this region and thus provide basis for designing prevention and control strategies, we investigated the serotypes and antibiotic resistance of the isolates obtained from the surveillance network during 2013 and 2018.

## Results

### Sample information

A total of 5239 cases (2749 of whom were male) were included during study period. Among these patients, 3870, 425, and 944 cases were enrolled from PKUSZH, SCH, and SYSU8H, respectively. SCH and SYSU8H started participating in the surveillance in 2016 and SCH was only involved in 2016. Patients ranged in age from 0 to 96 years (median 30 years). Local population who have registered permanent residence accounted for 60.4% of the patients. Of all the patients, 310 (5.9%) had fever, 635 (12.1%) had vomiting, and 50 (1.0%) had blood in stools, respectively. Of the patients over 5 years old, 2098 (54.1%) had abdominal pain (Table 1). Overall, *Salmonella* was isolated from 297 (5.7%) of all cases. The recovery rate in SCH, PKUSZH, and SYSU8H was 13.6%, 4.3%, and 7.7%, respectively.

**Table 1:** The epidemiological and clinical characteristics of samples (*n* = 5239) in this study

Category	Subcategory	No. (%)
Year	2013	931 (17.8)
	2014	793 (15.1)
	2015	652 (12.5)
	2016	1285 (24.5)
	2017	887 (16.9)
	2018	691 (13.2)
Age (years)	< 5	1361 (26.0)
	5 ~ 9	73 (1.4)
	10 ~ 19	217 (4.1)
	20 ~ 29	847 (16.2)
	30 ~ 39	984 (18.8)
	40 ~ 49	683 (13.0)
	50 ~ 59	496 (9.5)
	> = 60	578 (11.0)
Clinical Symptoms	Abdominal pain	2098 (54.1) ^a^
	Fever	310 (5.9)
	Vomiting	635 (12.1)
	Blood in stools	50 (1.0)

^a^Only 3878 cases aged over 5 years were included for analysis

### Serotyping results

A total of 42 serotypes were identified in the 285 *Salmonella* isolates and additional 12 strains were un-typable. Typhimurium (118, 39.7%) was the most common serotype, followed by serotype Enteritidis (71, 23.9%), London (12, 4.0%), 4, 5, 12: i: - (11, 3.7%), and Senftenberg (8, 2.7%). A total of 46 isolates with uncommon serotypes were found, including serotype Virchow, Corvallis, Vilvoorde, and Sarajane (Table 2). A high recovery rate (4.5%) of *S.* Typhimurium was observed in 2016 and onwards.

**Table 2:** The serotype distribution of clinical *Salmonella* isolates during 2013 and 2018

Serotype	No. of isolates by year (recovery rate, %)						
	2013 (*n* = 931)	2014 (*n* = 793)	2015 (*n* = 652)	2016 (*n* = 1285)	2017 (*n* = 887)	2018 (*n* = 691)	Total (*n* = 5239)
Typhimurium	6 (0.6)	8 (1.0)	5 (0.8)	58 (4.5)	22 (2.5)	19 (2.7)	118 (2.3)
Enteritidis	18 (1.9)	6 (0.8)	10 (1.5)	12 (0.9)	13 (1.5)	12 (1.7)	71 (1.4)
London	2 (0.2)	3 (0.4)	2 (0.3)	1 (0.1)	4 (0.5)	0 (0.0)	12 (0.2)
4, 5, 12: i: -	0 (0.0)	3 (0.4)	0 (0.0)	6 (0.5)	0 (0.0)	2 (0.3)	11 (0.2)
Senftenberg	1 (0.1)	6 (0.8)	0 (0.0)	0 (0.0)	0 (0.0)	1 (0.1)	8 (0.2)
Stanley	0 (0.0)	3 (0.4)	0 (0.0)	1 (0.1)	1 (0.1)	2 (0.3)	7 (0.1)
Agona	3 (0.3)	1 (0.1)	0 (0.0)	0 (0.0)	0 (0.0)	1 (0.1)	5 (0.1)
Litchfield	0 (0.0)	0 (0.0)	5 (0.8)	0 (0.0)	0 (0.0)	0 (0.0)	5 (0.1)
Weltevreden	3 (0.3)	0 (0.0)	0 (0.0)	0 (0.0)	0 (0.0)	1 (0.1)	4 (0.1)
Others	6 ^a^ (0.6)	2 ^b^ (0.3)	11 ^c^ (1.7)	7 ^d^ (0.5)	10 ^e^ (1.1)	8 ^f^ (1.2)	44 (0.8)
Un-typable	4 (0.4)	1 (0.1)	1 (0.2)	2 (0.2)	3 (0.3)	1 (0.1)	12 (0.2)
Total	43 (4.6)	33 (4.2)	34 (5.2)	87 (6.8)	53 (6.0)	47 (6.8)	297 (5.7)

^a ^Two strains of serotype Derby, 1 Ruzizi, 1 Meleagridis, and 2 Regent were included.

^b ^One isolate of serotype Gallinarum-pullorum and 1 Drogana were included.

^c ^One strain of Essen, 2 Manchester, 1 Sinstorf, 1 Chester, 1 Chomedey, 1 Tshiongwe, 1 Chennai, 1 Rissen, 1 Papuana, and 1 Fillmore were included.

^d ^Two strains of serotype Virchow, 2 Nigeria, 1 Bovismorbificans, 1 Hidalgo, and 1 Amherstiana were included.

^e ^Two strains of serotype Infantis, 1 Montevideo, 1 Bovismorbificans, 1 Chester, 2 Braenderup, 1 Kottbus, 1 Corvallis, and 1 Kentucky were included.

^f ^One strain of serotype Rissen, 1 Hato, 1 Sarajane, 1 Chester, 1 Assinie, 1 Pomona, 1 Muenster, and 1 Vilvoorde were included.

### Age and monthly distribution

The highest detection rate was observed in the age group of 5 ~ 9 years (15.1%), followed by < 5 years (10.1%) and 30 ~ 39 years (4.9%). The age group of over 60 years showed the lowest prevalence (2.8%). A high prevalence of *S.* Typhimurium infection was seen in the young children aged less than 5 years (6.4%) and 5 ~ 9 years old (5.5%). In the age group of 5 ~ 9 years, serotype Enteritidis and 4, 5, 12: i: - were the other two common serotypes (Table 3).

**Table 3:** The serotype distribution of clinical *Salmonella* isolates in different age groups

Age group (year)	No. of tested	No. of isolates (prevalence, %)							Total (%)
		Serotype Typhimurium	Serotype Enteritidis	Serotype 4, 5, 12: i: -	Serotype London	Serotype Senftenberg	Serotype Stanley	Other serotypes	
< 5	1361	87 (6.4)	17 (1.2)	8 (0.6)	5 (0.4)	1 (0.1)	4 (0.3)	15 (1.1)	137 (10.1)
5 ~ 9	73	4 (5.5)	6 (8.2)	1 (1.4)	0 (0.0)	0 (0.0)	0 (0.0)	0 (0.0)	11 (15.1)
10 ~ 19	217	0 (0.0)	4 (1.8)	0 (0.0)	1 (0.5)	1 (0.5)	0 (0.0)	3 (1.4)	9 (4.1)
20 ~ 29	847	7 (0.8)	14 (1.7)	0 (0.0)	0 (0.0)	3 (0.4)	1 (0.1)	11 (1.3)	36 (4.3)
30 ~ 39	984	9 (0.9)	14 (1.4)	0 (0.0)	1 (0.1)	2 (0.2)	0 (0.0)	22 (2.2)	48 (4.9)
40 ~ 49	683	3 (0.4)	4 (0.6)	1 (0.1)	3 (0.4)	1 (0.1)	1 (0.1)	11 (1.6)	24 (3.5)
50 ~ 59	496	3 (0.6)	6 (1.2)	1 (0.2)	0 (0.0)	0 (0.0)	0 (0.0)	6 (1.2)	16 (3.2)
> = 60	578	5 (0.9)	6 (1.0)	0 (0.0)	2 (0.3)	0 (0.0)	1 (0.2)	2 (0.3)	16 (2.8)
total	5239	118 (2.3)	71 (1.4)	11 (0.2)	12 (0.2)	8 (0.2)	7 (0.1)	70 (1.3)	297 (5.7)

Of the 118 *S.* Typhimurium isolates, 87 (73.7%) were recovered from children aged below 5 years old. Eight of 11 serotype 4, 5, 12: i: - strains were isolated from this age group, while similar detection rate of *S.* Enteritidis strains was observed in each age group. Of the 72 isolates with other serotypes, 51 (70.8%) were isolated from adults aged between 20 and 59 years old (Table 3).

**Fig. 1:**
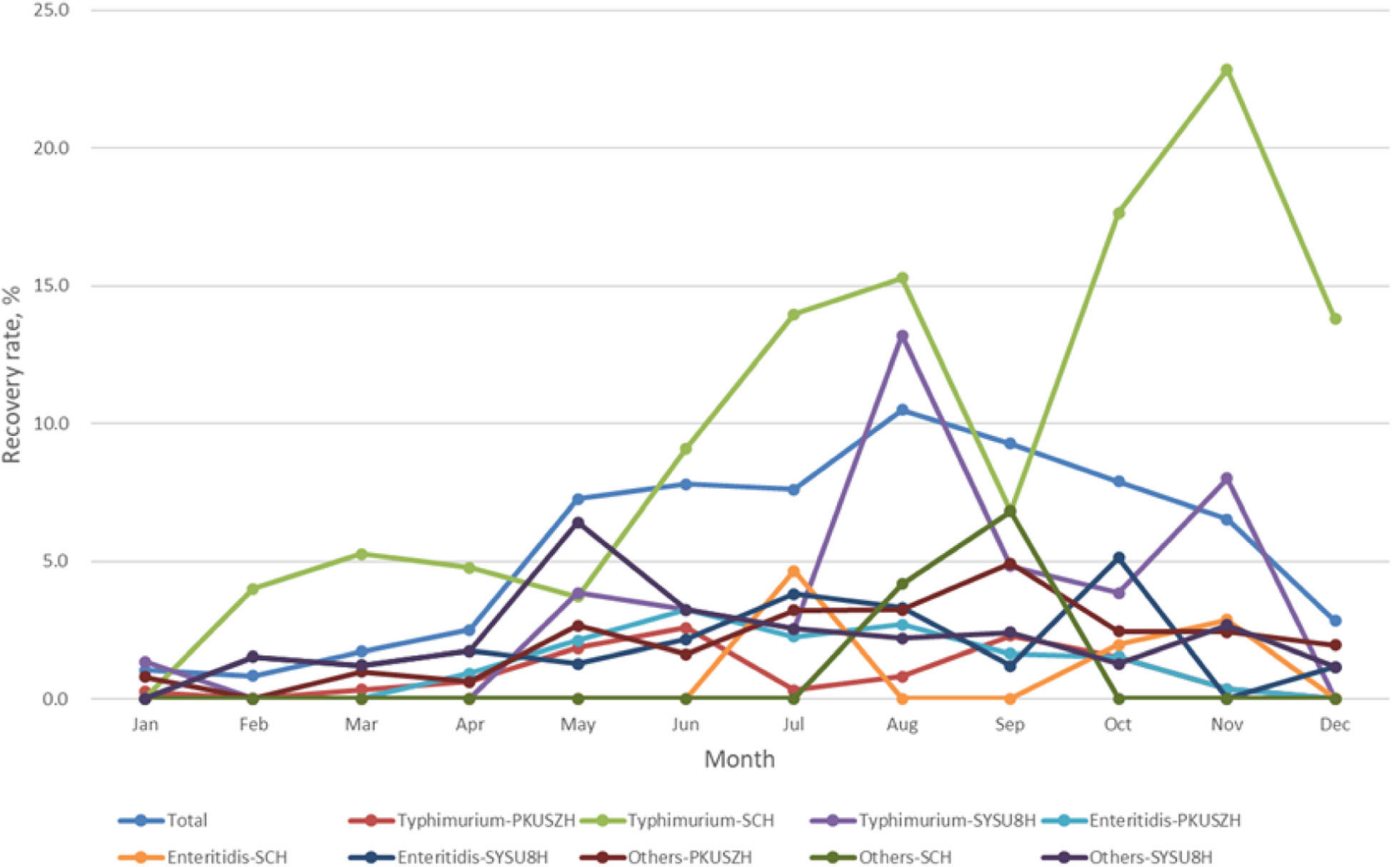
The monthly distribution of clinical *Salmonella* isolates. PKUSZH, Peking University Shenzhen Hospital; SCH, Shenzhen Children’s Hospital; SYSU8H, The Eighth Affiliated Hospital, Sun Yat-Sen University

The highest detection rate was seen in August (11.01%), followed by September (8.56%), June (8.49%), and October (7.88%). The detection rate between May and November (8.19%) was significantly higher than that between December and April (1.89%) (χ^2^ = 93.440, *P* < 0.001). Similar seasonal distribution was also observed in the serotype Typhimurium isolates (Fig. 1).

### Antimicrobial resistance profile

The highest rate of resistance was found in ampicillin (139, 70.6%), followed by piperacillin (127, 64.5%), tetracycline (125, 63.5%), streptomycin (107, 54.3%), cefazolin (87, 44.2%), and sulphamethoxazole/trimethoprim (75, 38.1%). The resistance to ampicillin and tetracycline was observed in 81 (95.3%) of *S.* Typhimurium isolates. Twenty-seven (93.1%) of *S.* Enteritidis isolates were resistant to nalidixic acid (Table 4). Five out of 8 *S.* Senftenberg isolates were susceptible to all the tested antibiotics. Among the 163 isolates resistant to three or more antimicrobial agents, 82 and 23 were found in *S.* Typhimurium and *S.* Enteritidis isolates, respectively. The multiple antibiotic resistance (MAR) index of 123 isolates was over 0.21. The highest MAR index (0.71) was found in a serogroup B un-typable strain.

**Table 4:** The antimicrobial susceptibility profiles of *Salmonella* isolates with different serotypes

Antimicrobial agent	No. of resistant isolates by serotypes (resistant rate, %)					
	Typhimurium (*n* = 85)	Enteritidis (*n* = 29)	London (*n* = 5)	Senftenberg (*n* = 8)	Others (*n* = 70)	Total (*n* = 197)
**Penicillins**						
ampicillin	81 (95.3)	22 (75.9)	2 (40.0)	0 (0.0)	34 (48.6)	139 (70.6)
piperacillin	74 (87.1)	21 (72.4)	2 (40.0)	0 (0.0)	30 (42.9)	127 (64.5)
**β-lactam/β-lactamase inhibitors**						
ampicillin/ sulbactam	9 (10.6)	7 (24.1)	0 (0.0)	1 (12.5)	6 (8.6)	23 (11.7)
**Cephems**						
cefazolin	50 (58.8)	16 (55.2)	1 (20.0)	1 (12.5)	19 (27.1)	87 (44.2)
cefepime	23 (27.1)	5 (17.2)	0 (0.0)	0 (0.0)	9 (12.9)	37 (18.8)
cefotaxime	40 (47.1)	8 (27.6)	0 (0.0)	1 (12.5)	15 (21.4)	64 (32.5)
ceftriaxone	42 (49.4)	9 (31.0)	0 (0.0)	1 (12.5)	14 (20.0)	66 (33.5)
cefoxitin	3 (3.5)	2 (6.9)	0 (0.0)	1 (12.5)	1 (1.4)	7 (3.6)
ceftazidime	13 (15.3)	3 (10.3)	0 (0.0)	0 (0.0)	7 (10.0)	23 (11.7)
**Monobactams**						
aztreonam	23 (27.1)	3 (10.3)	0 (0.0)	0 (0.0)	9 (12.9)	35 (17.8)
**Aminoglycosides**						
gentamicin	22 (25.9)	0 (0.0)	3 (60.0)	0 (0.0)	13 (18.6)	38 (19.3)
amikacin	2 (2.4)	0 (0.0)	0 (0.0)	0 (0.0)	0 (0.0)	2 (1.0)
streptomycin	57 (67.1)	20 (69.0)	2 (40.0)	1 (12.5)	27 (38.6)	107 (54.3)
**Carbapenems**						
imipenem	3 (3.5)	0 (0.0)	0 (0.0)	0 (0.0)	1 (1.4)	4 (2.0)
meropenem	2 (2.4)	0 (0.0)	0 (0.0)	0 (0.0)	0 (0.0)	2 (1.0)
**Tetracyclines**						
tetracycline	81 (95.3)	3 (10.3)	2 (40.0)	0 (0.0)	39 (55.7)	125 (63.5)
**Quinolones and Fluoroquinolones**						
ciprofloxacin	7 (8.2)	2 (6.9)	2 (40.0)	0 (0.0)	8 (11.4)	19 (9.6)
levofloxacin	5 (5.9)	1 (3.4)	0 (0.0)	0 (0.0)	6 (8.6)	12 (6.1)
norfloxacin	7 (8.2)	0 (0.0)	0 (0.0)	0 (0.0)	3 (4.3)	10 (5.1)
nalidixic acid	26 (30.6)	27 (93.1)	3 (60.0)	0 (0.0)	18 (25.7)	74 (37.6)
**Folate pathway inhibitors**						
sulphamethoxazole/trimethoprim	42 (49.4)	4 (13.8)	2 (40.0)	1 (12.5)	26 (37.1)	75 (38.1)
trimethoprim	38 (44.7)	3 (10.3)	2 (40.0)	0 (0.0)	22 (31.4)	65 (33.0)
**Phenicols**						
Chloramphenicol	41 (48.2)	2 (6.9)	1 (20.0)	0 (0.0)	21 (30.0)	65 (33.0)
**Nitrofurans**						
nitrofurantoin	2 (2.4)	11 (37.9)	0 (0.0)	0 (0.0)	8 (11.4)	21 (10.7)
**MAR index**						
<0.08	0 (0.0)	3 (10.3)	2 (40.0)	6 (72.5)	23 (32.9)	34 (17.3)
0.08-	3 (3.5)	3 (10.3)	0 (0.0)	1 (12.5)	5 (7.1)	12 (6.1)
0.13-	5 (5.9)	1 (3.4)	1 (20.0)	0 (0.0)	3 (4.3)	10 (5.1)
0.17-	10 (11.8)	2 (6.9)	0 (0.0)	1 (12.5)	5 (7.1)	18 (9.1)
≥0.21	67 (78.8)	20 (69.0)	2 (40.0)	0 (0.0)	34 (48.6)	123 (62.4)

Among the common serotypes, the lowest resistant rate was seen in *S.* Senftenberg. A significant higher frequency of resistance to penicillins, cephems (except for cefoxitin and ceftazidime), monobactams, tetracyclines, folate pathway inhibitors, and phenicols was observed in *S.* Typhimurium compared with that in other serotypes (*P* < 0.05), while resistant rate to nalidixic acid (*χ*^*2*^ = 45.227, *P* < 0.001) and nitrofurantoin (*χ*^*2*^ = 28.897, *P* < 0.001) was significantly higher in *S.* Enteritidis. Resistance to third generation cephalosporins and carbapenems was not found, while a higher resistant rate to ciprofloxacin and gentamicin was seen in *S.* London, compared with that in other serotypes (Table 4).

### Mutations with QRDRs

The amino acid alterations in *gyrA* occurred at codon 87 (Asp-87 → Gly or Asn) in 14 isolates and the MIC to CIP in 9 out of 14 isolates was over 2 μg/ml. The mutation in *gyrB* occurred at codon 462 and 464, but no amino acid alteration was found (Leu-462 → Leu, Ser-464 → Ser)*.* A single base change in amino acids 67, 75, 77, 117, and 123 in *parC* were found in 5 of the tested strains. The mutation of codons 500 and 509 in *parE* was found in 3 and 8 isolates, respectively (Table 5).

**Table 5:** The linkage of QRDRs mutations with antimicrobial susceptibility profile

Isolate No.	Serotype	MAR	MIC (μg/ml)		Mutations-changes in codons			
			CIP	LEV	*gyrA*	*gyrB*	*parC*	*parE*
1	Typhimurium	0.33	<=0.25	<=0.12	–	–	–	–
2	Typhimurium	0.46	0.5	0.5	–	–	–	–
3	Typhimurium	0.63	1	2	–	CTT → CT**C,** TCC → TC**T**^c^	GTT → GT**C,** CAC → CA**T,** CAT→CA**C,** GCG → GC**A,** TCC → TC**T**^d^	ACT→AC**G**^e^, CAC → CA**T**^f^
4	Bovismorbificans	0.38	1	0.5	GAC → G**G**C^a^	CTT → CT**C,** TCC → TC**T**	–	ACT, CAC → CA**T**
5	Typhimurium	0.50	1	0.5	GAC → G**G**C	CTT → CT**C,** TCC → TC**T**	–	ACT, CAC → CA**T**
6	Enteritidis	0.33	0.5	2	–	CTT → CT**C,** TCC → TC**T**	–	ACT, CAC → CA**T**
7	London	0.33	1	1	–	CTT → CT**C,** TCC → TC**T**	GTT → GT**C,** CAC → CA**T,** CAT→CA**C,** GCG → GC**A,** TCC → TC**T**	ACT, CAC → CA**T**
8	Typhimurium	0.33	2	1	GAC → **A**AC^b^	–	–	–
9	Agona	0.63	1	1	–	CTT → CT**C,** TCC → TC**T**	GTT → GT**C,** CAC → CA**T,** CAT→CA**C,** GCG → GC**A,** TCC → TC**T**	ACT→AC**G**, CAC → CA**T**
10	London	0.42	1	1	GAC → **A**AC	CTT → CT**C,** TCC → TC**T**	GTT → GT**C,** CAC → CA**T,** CAT→CA**C,** GCG → GC**A,** TCC → TC**T**	–
11	Litchfield	0.25	1	1	GAC → **A**AC	CTT → CT**C,** TCC → TC**T**	–	–
12	4, 5, 12, i: -	0.21	1	1	–	–	–	–
13	Enteritidis	0.08	1	1	GAC → **A**AC	CTT → CT**C,** TCC → TC**T**	–	ACT, CAC → CA**T**
14	London	0.13	2	0.5	GAC → **A**AC	CTT → CT**C,** TCC → TC**T**	GTT → GT**C,** CAC → CA**T,** CAT→CA**C,** GCG → GC**A,** TCC → TC**T**	ACT→AC**G**, CAC → CA**T**
15	4, 5, 12, i: -	0.50	2	2	GAC → **A**AC	–	–	–
16	untypable	0.42	2	4	GAC → **A**AC	–	–	–
17	4, 5, 12, i: -	0.42	> = 4	4	GAC → **A**AC	–	–	–
18	Typhimurium	0.42	2	2	GAC → **A**AC	–	–	–
19	Typhimurium	0.63	> = 4	> = 8	GAC → **A**AC	–	–	–
20	Typhimurium	0.46	2	4	GAC → **A**AC	–	–	–
21	Typhimurium	0.58	> = 4	4	GAC → **A**AC	–	–	–

Base pair changes in bold type.

MIC minimum inhibitory concentration, CIP ciprofloxacin, LEV levofloxacin.

- No mutation

^a^Amino acid alteration is Asp-87 → Gly.

^b^Amino acid alteration is Asp-87 → Asn.

^c^Amino acid alterations are Leu-462 → Leu, Ser-464 → Ser.

^d^Amino acid alterations are Val-67 → Val, His-75 → His, His-77 → His, Ala-117 → Ala, Ser-123 → Ser.

^e^Amino acid alteration is Thr-500 → Thr.

^f^Amino acid alteration is His-509 → His.

## Discussion

*Salmonella* was an important causative microorganism of acute gastroenteritis, which was shown in the detection rate during 2013 and 2018 (4.2% ~ 6.8%). The detection rate was lower compared with that in our previous study (12.1%) which incorporated PCR method [19], but it was comparable to another study (4.8%) conducted in Guangzhou using conventional assay [20]. A detection rate of 4.5% was reported in a large laboratory-based surveillance study in Guangdong province during 2009 and 2012, suggesting the continued prevalence in this region [17].

A broad distribution of serotypes was also reported in another study conducted in Shenzhen [21]. Apart from the detection rate, this study provided the trend of prevalence and antimicrobial resistance data over a long period, and identified some rare serotypes.

The detection rate between May and November was significantly higher compared with that in other months, which might be associated with high temperature. Hot weather contributed to bacterial growth and the chance of consumption of insufficiently heat-treated food or salad was higher. A linear association between temperature and the number of reported cases of salmonellosis was found and it was proven that higher temperature was associated with *Salmonella* infections [22]. As a result, the sporadic cases and salmonellosis outbreak were commonly found during this period [17].

The high incidence of *Salmonella* infection in children was consistent with another study where a recovery rate of 17.2% in children was reported [15]. The high frequency of infections in children could be attributed to the behavior such as frequent contact with contaminated limbs, consumption of contaminated food or water, or close contacts with an asymptomatic caretaker [20]. *S.* Typhimurium and *S.* Enteritidis were the most common serotypes in children aged below 5 years old, which represented 75.4% (104/138) of the isolates, in accordance with other studies [15, 17]. The high occurrence of *S.* Typhimurium in the age group of < 5 years and 5 ~ 9 years might be associated with relatively low immune response and behaviors of these young children [23]. Highest recovery rate and highest occurrence of *S.* Enteritidis in the age group of 5 ~ 9 years was probably due to the small sample size. The high frequency of multi-drug resistance in *S.* Typhimurium posed the difficulties in treating pediatric patients. Some measures such as good hygiene, proper hand washing, and education of their guardians were recommended to reduce the disease burden of salmonellosis in children.

Wide distribution of serotypes might be another reason of the continued prevalence of *Salmonella* infections*.* Apart from *S.* Typhimurium and *S.* Enteritidis, a broad range of uncommon serotypes including Vilvoorde and Sarajane were identified, indicating the wide sources of *Salmonella*. As one of the major pathogenic bacteria in Chinese food commodities [24], *Salmonella* was commonly isolated from beef, pork, and poultry meat [25, 26]. Agona, Corvallis, and Kentucky were reported the dominant serotypes in chicken samples, while Typhimurium, Rissen, and Derby were the most common in pork samples [16]. The dominant presence of *S.* Typhimurium in 2016 and onwards was attributed to the participation of SCH and SYSU8H after 2016. Thirty-six of *S.* Typhimurium strains were isolated from PKUSZH, while 82 isolates were from the other two hospitals.

Serotype Gallinarum-pullorum was isolated from a 24-year-old male patient with mild diarrhea. *S*. Gallinarum-pullorum was generally regarded as chicken-derived which caused little public health concern. However, occasional infections in human were reported following consumption of heavily contaminated food and low detection rate was found in humans between 1982 and 1992 [27]. Transient illness caused by large number of *S*. Gallinarum-pullorum was observed in both volunteers [28] and our case. The contaminated raw meat was not considered as a food safety risk due to the thorough cooking tradition, but it was often associated with direct exposure to enteric pathogens and cross-contamination of ready-to-eat foods [29]. As a result, contamination from food handlers, or the consumption of contaminated or cross contaminated food may lead to the *Salmonella* infections.

*Salmonella* infections are normally associated with self-limiting diarrhea and antimicrobial therapy is not indicated, but appropriate antimicrobial treatment could be life-saving in severe cases. In addition, antibiotic treatment with ciprofloxacin or fluoroquinolones was recommended for *Salmonella* infections in infants less than 3 months of age due to the high risk of bacteremia and extraintestinal complications [30, 31]. The occurrence of multiple drug resistant strains raised the importance of antibiotics resistance surveillance. The high level of resistance to the first-line agents: ampicillin, sulphamethoxazole/trimethoprim, and chloramphenicol was also observed in other studies [20]. A similar profile was also found in the isolates from food-producing animals [32], suggesting the careful use of antibiotics in breeding industry was necessary.

The frequency of multiple drug resistance was common. Resistance to 5 or more antibiotics was commonly observed in *S.* Enteritidis (69.0%) and *S.* Typhimurium (78.8%). The occurrence of multidrug resistant (MDR) strains was reported to be associated with coexistence of resistance-related genes [14] and their transmission led to great difficulties in treatments. The resistance to first-line drugs of treating severe infections, such as third-generation cephalosporins and fluoroquinolones was of clinical concerns. The emergence of MDR isolates and increasing resistance to important antibiotics suggested the prevention measures and ongoing surveillance of antibiotic resistance are needed to control the infections.

The resistance to ciprofloxacin was mediated by multiple mechanisms [33], and the main mechanism of mutations in QRDRs was investigated in this study. A high frequency of mutations in *gyrA*, *gyrB*, *parC*, and *parE* genes were found in the quinolone-resistant strains. The amino acid alterations in *gyrA* was associated with high levels of MIC to CIP and LEV, suggesting that the mutations in *gyrA* played an important role in the drug resistance. It was also reported in other studies that mutations associated with quinolone resistance were mainly present in the QRDRs of *gyrA* gene [34, 35], probably due to that the DNA gyrase was the primary target of quinolone action and a single point mutation of *gyrA* could lead to the reduced susceptibility to fluoroquinolones. In the isolates absence of chromosomal mutations where no amino acid alteration was found, other mechanisms such as over-expression of efflux pump and mutations of other elements might contribute to the resistance [36, 37].

## Conclusion

1. *Salmonella* was an important causative microorganism of acute diarrhea in this region and a broad range of serotypes were prevalent.
2. *S.* Typhimurium and *S.* Enteritidis were the two most common serotypes. The highest detection rate was found in the age group of less than 9 years old and during June and October.
3. A high rate of MDR was found in serotype Typhimurium and Enteritidis. An increasing trend of resistant rate to fluoroquinolones was mainly associated with the point mutation in the QRDRs of *gyrA* gene.

## Methods

### Stool samples collection

Peking University Shenzhen Hospital (PKUSZH), Shenzhen Children’s Hospital (SCH), and The Eighth Affiliated Hospital, Sun Yat-Sen University (SYSU8H) were selected as sentinel hospitals in this retrospective study. Stool samples were collected from outpatients who visited gastroenteritis clinic due to acute infectious diarrhea and agreed to take part in the surveillance program. Acute diarrhea was defined as over 3 passages of loose, mucus-, watery, or bloody-stools during 24-h period. The stool samples were examined for *Salmonella* sp. using CHROM agars. The clinical signs and demographic information were retrospectively collected from electronic medical records.

### Serotyping and antimicrobial susceptibility testing

The stool specimens were enriched in selenite cysteine (SC) broth and then plated on CHROM agar for isolation of *Salmonella* spp.. The suspicious colonies were identified using Vitek-2 compact system (bioMerieux, France) and *Salmonella* spp. isolates were serotyped with a commercial serotyping kit (S&A company, S&A Reagent Lab, Bangkok, Thailand) in the sentinel hospitals. Then part of the isolates were sent to Futian District Center for Disease Control and Prevention (CDC) and Shenzhen Hospital, Southern Medical University and stored in − 80 °C for further analysis.

A total of 197 collected *Salmonella* isolates representing 26 serotypes were recovered and tested for antimicrobial susceptibility in 2019. Twenty-four antimicrobials (Oxoid, UK) including amikacin (30 micrograms (mcg), AK), ampicillin/sulbactam (20 mcg, SAM), ampicillin (10mcg, AMP), aztreonam (30 mcg, ATM), cefepime (30 mcg, FEP), cefotaxime (30 mcg, CTX), cefoxitin (30 mcg, FOX), ceftazidime (30 mcg, CAZ), ceftriaxone (30 mcg, CRO), cephazolin (30 mcg, KZ), chloramphenicol (30 mcg, C), ciprofloxacin (5 mcg, CIP), gentamicin (10 mcg, CN), imipenem (10 mcg, IPM), levofloxacin (5 mcg, LEV), meropenem (10 mcg, MEM), nalidixic acid (30 mcg, NA), nitrofurantoin (300 mcg, F), norfloxacin (10 mcg, NOR), piperacillin (100 mcg, PRL), streptomycin (10 mcg, S), sulphamethoxazole/trimethoprim (25 mcg, SXT), tetracycline (30 mcg, TE), and trimethoprim (5 mcg, W) were tested using disk diffusion method. The susceptibility to CIP and LEV in quinolone-resistant isolates was confirmed by Vitek-2 compact system (bioMerieux, France).

The MacFarland 0.5 inoculums were prepared and swabbed on the entire surface of Mueller-Hinton agar (Huankai, China) and left to dry for 3–5 min. Antimicrobial susceptibility test discs (Oxoid, UK) were placed on the inoculated agar plate with a disc dispenser (Oxoid, UK) and incubated at 37 °C for 24 h. After incubation, the diameter of inhibition zone was measured and the results were interpreted as susceptible, intermediate, and resistant according to the Clinical and Laboratory Standards Institute guideline (CLSI, 2019) [38]. *Escherichia coli* strain ATCC 25922 and *Pseudomonas aeruginosa* strain ATCC 27853 was used as quality control for disk diffusion and Vitek-2 compact system, respectively.

The multiple antibiotic resistance (MAR) index was the ratio between the number of antibiotics to which the organism was resistant and the number of antibiotics tested. Multi-drug resistant (MDR) was defined as resistant to three or more different classes of antimicrobial agents. Statistical analysis was conducted using chi-square test by SPSS version 21 (SPSS Inc., Chicago, IL, USA).

### Detection of target gene mutations

A total of 21 isolates that resistant to any of the three antibiotics: ciprofloxacin, levofloxacin, and norfloxacin were chosen to screen for the mutations of the *gyrA, gyrB, parC,* and *parE* genes in the QRDRs. The bacterial nucleic acid was extracted using QIAamp DNA Mini Kit (Qiagen GmbH, Hilden, Germany) and subjected to PCR amplification using *Taq* PCR Master Mix Kit (Qiagen GmbH, Hilden, Germany), according to the manufacturer’s recommended protocols. The PCR products were sent to Sangon (Shanghai, China) for sequencing and the results were analyzed using BLAST (PubMed). The primers used for PCR amplification and sequencing were listed in Supplementary Table 1 [39, 40].

## Supplementary information

**Additional file 1: Table S1.** List of primers used in this study.

## Data Availability

The datasets used and analyzed during the current study are available from the corresponding authors on reasonable request.

## Abbreviations

AMP: Ampicillin
AK: Amikacin
ATM: Aztreonam
C: Chloramphenicol
CAZ: Ceftazidime
CDC: Center for Disease Control and Prevention
CIP: Ciprofloxacin
CLSI: Clinical and Laboratory Standards Institute
CN: Gentamicin
CRO: Ceftriaxone
CTX: Cefotaxime
F: Nitrofurantoin
FEP: Cefepime
FOX: Cefoxitin
IPM: Impenem
KZ: Cephazolin
LEV: Levofloxacin
MAR: Multiple antibiotic resistance
mcg: Micrograms
MDR: Multi-drug resistant
MEM: Meropenem
MIC: Minimum inhibitory concentration
NA: Nalidixic acid
NOR: Norfloxacin
PCR: Polymerase chain reaction
PKUSZH: Peking University Shenzhen Hospital
PRL: Piperacillin
QRDRs: Quinolone-resistance determining regions
S: Streptomycin
SAM: Ampicillin/sulbactam
SCH: Shenzhen Children’s Hospital
SXT: Sulphamethoxazole/trimethoprim
SYSU8H: The Eighth Affiliated Hospital, Sun Yat-Sen University
TE: Tetracycline
W: Trimethoprim
