# Supplementary material for: Prevalence, serotypes, and antimicrobial resistance of Salmonella isolates from patients with diarrhea in Shenzhen, China
Source: BMC Microbiol. 2020 Jul 6;20:197. doi: 10.1186/s12866-020-01886-5 (PMC7339465; doi:10.1186/s12866-020-01886-5)
Supplement: Supplementary file 1 — Additional file 1: Table S1. List of primers used in this study. [file 12866_2020_1886_MOESM1_ESM.docx]

Supplementary TABLE 1. List of primers used in this study

| Resistance genes | Primer (5’-3’) | Size (bp) | Reference |
| --- | --- | --- | --- |
| *gyrA* | F: AAATCTGCCCGTGTCGTTGGT | 344 | [21] |
|  | R: GCCATACCTACTGCGATACC |  |  |
| *gyrB* | F: GAATACCTGCTGGAAAACCCAT | 446 | [21] |
|  | R: CGGATGTGCGAGCCGTCGACGTCCGC |  |  |
| *parC* | F: AAGCCGGTACAGCGCCGCATC | 395 | [21] |
|  | R: GTGGTGCCGTTCAGCAGG |  |  |
| *parE* | F: TCTCTTCCGATGAAGTGCTG | 237 | [22] |
|  | R: ATACGGTATAGCGGCGGTAG |  |  |
